# Targeted Metabolite and Gene Expression Analysis of Anthocyanin and Kaempferol Glycoside Accumulation in Peach Accessions with Contrasting Flesh and Skin Pigmentation

**DOI:** 10.3390/foods15122225

**Published:** 2026-06-20

**Authors:** Weifeng Chen, Dan Tang, Jia Huang, Yu Yang, Liangbo Zhang

**Affiliations:** Hunan Horticultural Research Institute, Hunan Academy of Agricultural Sciences/Yuelushan Laboratory, Changsha, 410125, China; weifengchen88@hunaas.cn (W.C.); tangdan9393@163.com (D.T.); huangjia8820@163.com (J.H.)

**Keywords:** peach, anthocyanin, kaempferol glycoside, fruit pigmentation, *PpDFR*, *PpFLS*

## Abstract

Peach (*Prunus persica*) fruit pigmentation is largely associated with anthocyanin accumulation, whereas colorless flavonols such as kaempferol glycosides may reflect alternative use of shared flavonoid precursors. To examine the relationship between anthocyanin and selected kaempferol glycoside accumulation, we analyzed 15 peach accessions classified by red, white, or yellow flesh pigmentation. Skin color was quantified using the a*/b* ratio, where a* represents redness/greenness and b* represents yellowness/blueness. Red-fleshed accessions showed higher skin a*/b* values and accumulated higher levels of total anthocyanins, particularly cyanidin-3-glucoside, than white and yellow accessions. In contrast, kaempferol-3-rhamnoside preferentially accumulated in white-fleshed accessions. Expression analysis of flavonoid pathway genes showed that dihydroflavonol 4-reductase (*PpDFR*) was more highly expressed in red accessions, whereas flavonol synthase (*PpFLS*) was more highly expressed in white accessions; chalcone synthase (*PpCHS*), flavanone 3-hydroxylase (*PpF3H*), flavonoid 3′-hydroxylase (*PpF3′H*), and anthocyanidin synthase (*PpANS*) showed no significant differences among color groups. Heterologous overexpression of *PpF3′H* in *Arabidopsis thaliana*, a well-characterized model plant for flavonoid biosynthesis, was associated with increased seed anthocyanin accumulation and a lower kaempferol-to-quercetin ratio, supporting its catalytic capacity to influence flavonoid composition in an exogenous system. Overall, these results indicate that differential anthocyanin and selected kaempferol glycoside accumulation in peach is associated with the relative expression patterns of branch-related flavonoid genes, particularly *PpDFR* and *PpFLS*. This study provides targeted metabolic and transcriptional evidence for understanding peach flesh and skin pigmentation and provides mechanistic insight into flavonoid branch competition linking gene expression patterns with metabolite allocation, and identifies candidate genes for improving fruit color and flavonoid-related nutritional quality.

## 1. Introduction

Peach (*Prunus persica*) is widely appreciated not only for its appealing taste and aroma but also for its abundant health-promoting phytochemicals [1,2,3]. Among these phytochemicals, flavonoids, particularly anthocyanins and flavonols such as kaempferol, have attracted attention because of their physiological roles and potential nutritional value [4,5,6]. Because fruit coloration can visually reflect the accumulation of antioxidant phenolic compounds, understanding pigmentation-related flavonoid variation is relevant for both fruit-quality evaluation and breeding for enhanced nutritional attributes.

Anthocyanins are the major pigments responsible for red coloration and also function in photoprotection and reactive oxygen scavenging [3,7,8,9]. In contrast, flavonols such as kaempferol and quercetin are largely colorless compounds involved in stress responses and antioxidant activity [10,11,12]. Although they do not directly determine pigmentation, flavonols can influence anthocyanin stability through co-pigmentation effects [13].

**Figure 1:**
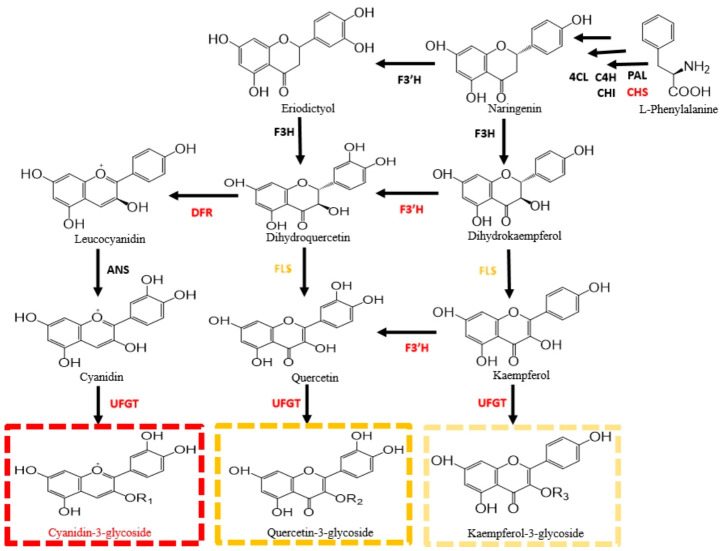
Simplified flavonoid biosynthetic pathway showing the anthocyanin, kaempferol glycoside, and quercetin glycoside branches. Red highlights indicate the anthocyanin branch, while yellow highlights indicate the kaempferol and quercetin glycoside branches. Key enzymes analyzed in this study include CHS, F3H, F3′H, FLS, DFR, ANS, and UDP-glucose:flavonoid 3-O-glucosyltransferase (UFGT). Arrows indicate the direction of metabolic conversion, and dashed boxes mark representative glycosylated end products. Abbreviations: PAL, phenylalanine ammonia-lyase; 4CL, 4-coumarate:CoA ligase; CHS, chalcone synthase; CHI, chalcone isomerase; F3H, flavanone 3-hydroxylase; F3′H, flavonoid 3′-hydroxylase; DFR, dihydroflavonol 4-reductase; FLS, flavonol synthase; ANS, anthocyanidin synthase; UFGT, UDP-glucose:flavonoid 3-O-glucosyltransferase.

In the flavonoid biosynthetic pathway, anthocyanins and flavonols originate from shared dihydroflavonol precursors [14,15]. At this branch point, DFR and FLS use related substrates and are therefore expected to influence the relative accumulation of anthocyanins and flavonols [16,17]. In addition, F3′H converts dihydrokaempferol into dihydroquercetin, thereby affecting the relative accumulation of kaempferol- and quercetin-derived compounds [18]. Because DFR and FLS differ in substrate preference, changes in gene expression and substrate availability may alter flavonoid composition [17,18,19]. A schematic representation of this pathway is provided in Figure 1 [14].

Previous studies in apple and other Rosaceae species suggest that anthocyanin and flavonol accumulation may be coordinated through shared flavonoid precursors [12,20]. However, whether a similar inverse association between anthocyanins and selected kaempferol glycosides occurs in peach fruit tissues remains insufficiently understood. Although several studies have investigated anthocyanin-related structural genes and transcription factors in peach, fewer studies have jointly evaluated target flavonoid metabolites and the expression of branch-related genes such as *PpDFR*, *PpFLS*, and *PpF3′H* across diverse peach accessions.

In this study, we performed targeted metabolite analysis and reverse transcription quantitative polymerase chain reaction (RT-qPCR)-based gene expression profiling of 15 peach accessions with contrasting flesh colors. We quantified total anthocyanins and selected anthocyanin and kaempferol glycoside compounds, analyzed the expression of major flavonoid biosynthetic genes (*PpCHS*, *PpF3H*, *PpF3′H*, *PpFLS*, *PpDFR*, and *PpANS*), and assessed the potential function of *PpF3′H* through heterologous expression in *Arabidopsis thaliana*. These genes were selected because they represent early pathway entry steps (*PpCHS* and *PpF3H*), 3′-hydroxylation (*PpF3′H*), branch-related flavonol and anthocyanin steps (*PpFLS* and *PpDFR*), and downstream anthocyanidin formation (*PpANS*). *PpF3′H* was selected for functional assessment because it affects the conversion of dihydrokaempferol to dihydroquercetin and may influence the relative abundance of kaempferol- and quercetin-derived compounds. We hypothesized that anthocyanin accumulation in red-fleshed peach accessions would be associated with higher *PpDFR* expression and lower selected kaempferol glycoside accumulation, whereas white-fleshed accessions would show relatively higher *PpFLS* expression and kaempferol glycoside accumulation.

## 2. Materials and Methods

### 2.1. Plant Materials

Three groups of peach accessions with different flesh colors at the mature stage were selected: five red-fleshed lines (‘7-3-58’, ‘7-3-64’, ‘6-11-50’, ‘Bei 22-31 Dong’, and ‘Bei 23-20 Xi’), five white-fleshed lines (‘08 Bei-8-4’, ‘Zhongyou *Mini* No. 1’, ‘Zhongyou 12’, ‘Xin Zhongnan 40-35’, and ‘Chunmi’), and five yellow-fleshed lines (‘Xin Xibei 57-61’, ‘Zhongyou 19’, ‘Xin Xinan 34-8’, ‘Xin Zhongnan 11-30’, and ‘Huangjinmi No. 7’). All fruits were obtained from the experimental base of the Zhengzhou Fruit Research Institute, Chinese Academy of Agricultural Sciences, under uniform orchard management. Fruits were sampled at comparable commercial maturity based on typical ripening characteristics on 8 June 2024. Mature peach skin and flesh samples were immediately frozen in liquid nitrogen, protected from light, and stored at −80 °C until extraction. Repeated freeze–thaw cycles were avoided. Three biological replicates were performed for each accession, with each replicate taken from a different individual tree. Detailed accession information and fruit quality indices, including fruit weight, soluble solids content, titratable acidity, and firmness, are provided in Supplementary Table S1.

### 2.2. Determination of L*, a*, and b* Values

Fruit color parameters (L*, a*, and b*) were measured using a CHROMA METER CR-400 colorimeter (Konica Minolta, Tokyo, Japan). Fully mature fruits were randomly selected, and three points were evenly measured on the fruit surface to obtain average L*, a*, and b* values. Three fruits per accession were measured, with three repeated readings per fruit. L* represents lightness (0 = black, 100 = white), a* represents the red-green axis (a* > 0, red; a* < 0, green), and b* represents the yellow-blue axis (b* > 0, yellow; b* < 0, blue). The a*/b* ratio was calculated to quantify the relative redness of the skin. L* values are provided in Supplementary Table S2.

### 2.3. Determination of Total Anthocyanin Content

Total anthocyanin content was determined according to Xie et al. [21]. Briefly, 100 mg each of frozen, ground peach skin, peach flesh, and *Arabidopsis* seeds were separately mixed with 1 mL of 1% (*v*/*v*) HCl-methanol extraction solution and incubated in the dark at room temperature for 24 h, with mixing 2–3 times during the incubation. After centrifugation at 13,000× g for 5 min, the absorbance of the supernatant was measured at 530 nm, 620 nm, and 650 nm using a UV-2600 UV–Vis spectrophotometer (Shimadzu Corporation, Kyoto, Japan).

Optical density of anthocyanins (δOD) = (A_530_ − A_620_) − 0.1 × (A_650_ − A_620_)

Total anthocyanin content (μg·g^−1^ fresh weight (FW)) = δOD/ε × V/M × 10^6^, where ε is the molar extinction coefficient at 530 nm (4.62 × 10^4^), V is the extraction volume (mL), and M is the sample mass (g).

### 2.4. Determination of Anthocyanins, Kaempferol Glycosides and Quercetin Glycosides

Target anthocyanins, kaempferol glycosides, and quercetin glycosides were extracted and analyzed using high-performance liquid chromatography (HPLC), following the protocol described by Li et al. [22] with minor modifications. Frozen peach peel tissue (0.5 g) was homogenized in 1.5 mL of an ice-cold extraction solvent composed of 70% methanol and 2% formic acid. The homogenate was centrifuged at 10,000× g for 20 min at 4 °C, and the resulting supernatant was filtered through a 0.22-μm syringe filter before instrumental analysis.

Authentic standards of cyanidin-3-glucoside, cyanidin-3-rutinoside, kaempferol-3-rhamnoside, and kaempferol-3-galactoside were purchased from Sigma-Aldrich (St. Louis, MO, USA). Calibration curves were constructed using serial dilutions of each standard, and compound concentrations were calculated using the external standard method.

Chromatographic separation was achieved on an Inertsil ODS-3 column (5 μm, 4.6 mm × 250 mm; GL Sciences Inc., Tokyo, Japan) protected by a guard column of the same material. The injection volume was 10 μL, and the column temperature was maintained at 35 °C. The mobile phase consisted of solvent A (10% formic acid in water) and solvent B (10% formic acid in acetonitrile, containing 1.36% water). The gradient elution program was set as follows: 0 min, 95% A; 25 min, 85% A; 42 min, 78% A; 60 min, 64% A; and 65 min, 95% A, followed by a 10 min post-run re-equilibration. The flow rate was kept constant at 1.0 mL/min. Detection was performed using a diode array detector (DAD), with kaempferol glycosides and quercetin glycosides monitored at 365 nm and anthocyanins at 520 nm. Peaks were identified by comparing retention times and UV absorption spectra with authentic standards, and concentrations were calculated from compound-specific calibration curves. Representative HPLC chromatograms and calibration curves for the detected anthocyanins and kaempferol glycosides are shown in Supplementary Figure S1.

### 2.5. RT-qPCR Analysis

Total RNA was extracted from peach skin and *Arabidopsis* inflorescences using a plant total RNA extraction kit (Takara Bio Inc., Kusatsu, Shiga, Japan) according to the manufacturer’s protocol. The concentration and purity of RNA were checked using a spectrophotometer, and complementary DNA (cDNA) was synthesized for RT-qPCR analysis. RT-qPCR was performed using SYBR Premix Ex Taq™ II (Takara Bio Inc., Kusatsu, Shiga, Japan) and a Bio-Rad CFX96 real-time PCR system (Bio-Rad Laboratories, Hercules, CA, USA). The reaction mixture (20 μL) contained 10 μL SYBR dye, 1 μL each of forward and reverse primers, 2 μL cDNA template, and 7 μL sterile double-distilled water (ddH2O). Three biological replicates were analyzed for each treatment. The PCR program consisted of 95 °C for 3 min, followed by 39 cycles of 95 °C for 10 s and 57 °C for 30 s, with a subsequent melt curve analysis. *PpActin* was used as the internal reference gene for peach samples, and *AtActin2* was used for *Arabidopsis* samples. Primers for all target and reference genes are listed in Supplementary Table S3.

### 2.6. Transformation of Arabidopsis with PpF3′H and Flavonoid Analysis

To assess the potential function of peach *PpF3′H* in a heterologous system, the *PpF3′H* coding sequence (GenBank: JQ697494.1) was amplified from cDNA of Huangjinmi No. 7 and cloned into the pCAMBIA2300 vector under the control of the cauliflower mosaic virus (CaMV) 35S promoter and introduced into wild-type *Arabidopsis thaliana* Col-0 via *Agrobacterium tumefaciens* strain GV3101 using the floral dip method (Figure S2) [23]. After the floral-dip transformation, 13 independent T0 transgenic lines were obtained under 50 mg/L kanamycin selection on Murashige and Skoog (MS) medium. Positive transformants were confirmed by polymerase chain reaction (PCR) using a 35S promoter forward primer and a *PpF3′H*-specific reverse primer, followed by sequencing. Three independent T2 lines were selected for further analysis. The expression level of *PpF3′H* was verified by RT-qPCR, and the overexpression (OE) line OE-1 showed consistent expression and flavonoid-related phenotypes. Transgenic and wild-type plants were grown side by side at 22 °C with 70% relative humidity under a 16 h light/8 h dark photoperiod. Flavonoid composition and total anthocyanin content in *Arabidopsis* tissues were analyzed as described in Section 2.3 and Section 2.4.

### 2.7. Statistical Analysis

Data were analyzed using SPSS 16.0 (SPSS Inc., Chicago, IL, USA). Normality and homogeneity of variance were assessed using Shapiro–Wilk and Levene’s tests, respectively. For comparisons among the red-, white-, and yellow-fleshed groups, one-way analysis of variance (ANOVA) followed by Tukey’s honestly significant difference (HSD) test was used. For comparisons between wild-type and *PpF3′H*-overexpressing *Arabidopsis* plants, a two-tailed Student’s t-test was used according to variance homogeneity. Statistical significance was set at *p* < 0.05 unless otherwise indicated. All statistical analyses were conducted based on at least three independent biological replicates (*n* ≥ 3), and data are presented as mean ± SD.

## 3. Results

### 3.1. Color Difference Analysis of Peach Skin in Accessions with Contrasting Flesh Colors

**Figure 2:**
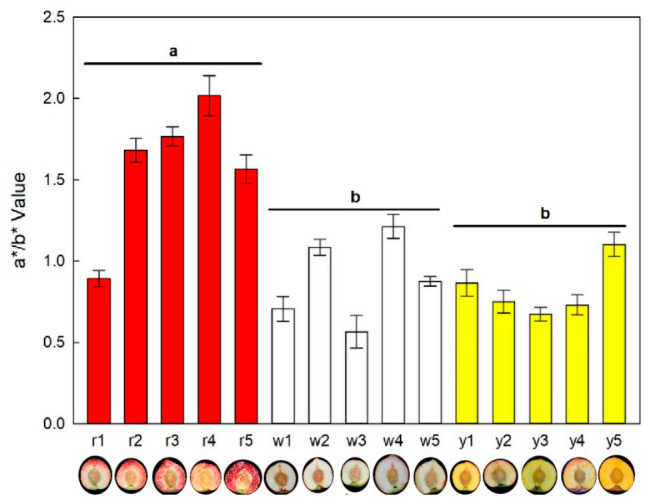
Coloration of peach skin in accessions with different flesh colors. Different lowercase letters indicate significant differences among color groups (one-way ANOVA followed by Tukey’s HSD test, *p* < 0.05). The horizontal line indicates the mean value of each color group. Note: The first letter of the sample code indicates the fruit flesh color: r, red; w, white; y, yellow. r1: ‘7-3-58’ (red), r2: ‘7-3-64’ (red), r3: ‘6-11-50’ (red), r4: ‘Bei 22-31 Dong’ (red), r5: ‘Bei 23-20 Xi’ (red); w1: ‘08 Bei-8-4’ (white), w2: ‘Zhongyou Mini No. 1’ (white), w3: ‘Zhongyou 12’ (white), w4: ‘Xin Zhongnan 40-35’ (white), w5: ‘Chunmi’ (white); y1: ‘Xin Xibei 57-61’ (yellow), y2: ‘Zhongyou 19’ (yellow), y3: ‘Xin Xinan 34-8’ (yellow), y4: ‘Xin Zhongnan 11-30’ (yellow), y5: ‘Huangjinmi No. 7’ (yellow).

To quantify skin color differences among peach accessions with contrasting flesh colors, L*, a*, and b* values were measured for 15 accessions (Figure 2; Supplementary Table S2). The a*/b* values of red-fleshed accessions (r) were significantly higher than those of white-fleshed (w) and yellow-fleshed (y) accessions. Among the red-fleshed accessions, r4 had the highest a*/b* value (2.02), while r1 had the lowest (0.89). Within the white-fleshed accessions, w2 (1.09) and w4 (1.21) showed relatively higher a*/b* values, whereas w3 exhibited the lowest value (0.57). For the yellow-fleshed accessions, y5 had the highest a*/b* value (1.10), and y3 had the lowest (0.67).

A higher a*/b* ratio indicates stronger red coloration of the skin. Red-fleshed peaches generally exhibited higher a*/b* values, suggesting partial consistency between flesh pigmentation and skin redness. The a*/b* values of white- and yellow-fleshed accessions partially overlapped, indicating genotype-specific variation in skin coloration. These results show that the a*/b* ratio is useful for describing the degree of skin redness, while flesh pigmentation and skin pigmentation should be treated as related but distinct traits.

### 3.2. Analysis of Anthocyanin Content with Different Colors

**Figure 3:**
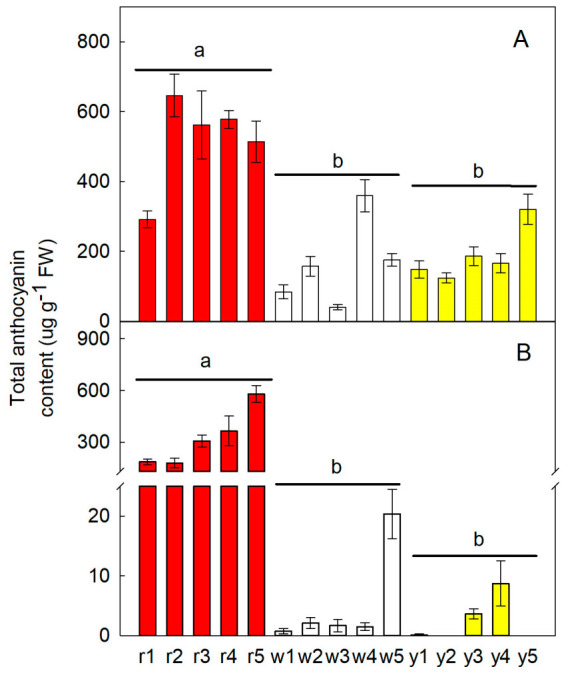
Anthocyanin content in peach accessions grouped by flesh pigmentation. (**A**) Skin; (**B**) flesh. Different lowercase letters indicate significant differences among color groups based on one-way ANOVA followed by Tukey’s HSD test (*p* < 0.05). The horizontal line indicates the mean value of each color group. Sample codes are as defined in Figure 2.

To clarify differences in anthocyanin accumulation among peach fruits with different flesh colors, total anthocyanin content was measured in the skin and flesh of 15 accessions (Figure 3). In the skin, all accessions accumulated detectable anthocyanins, with red-fleshed accessions (r) exhibiting significantly higher levels than white-fleshed (w) and yellow-fleshed (y) accessions. The skin of r2 showed the highest anthocyanin content (646.88 μg g^−1^ FW), which was approximately 7.7-fold higher than that of w1 and 4.4-fold higher than that of y1 (Figure 3A). In the flesh, red-fleshed accessions also accumulated significantly higher anthocyanin levels than white- and yellow-fleshed accessions, with r5 having the highest content (579.72 μg g^−1^ FW). Most white- and yellow-fleshed accessions had extremely low or undetectable anthocyanin levels in the flesh, with only w5 (20.35 μg g^−1^ FW) and y4 (8.74 μg g^−1^ FW) showing minor accumulation (Figure 3B). These results suggest that red-fleshed peaches have greater anthocyanin accumulation, which is consistent with their higher skin a*/b* values.

### 3.3. Targeted Analysis of Anthocyanins and Kaempferol Glycosides in Peach Skin

**Figure 4:**
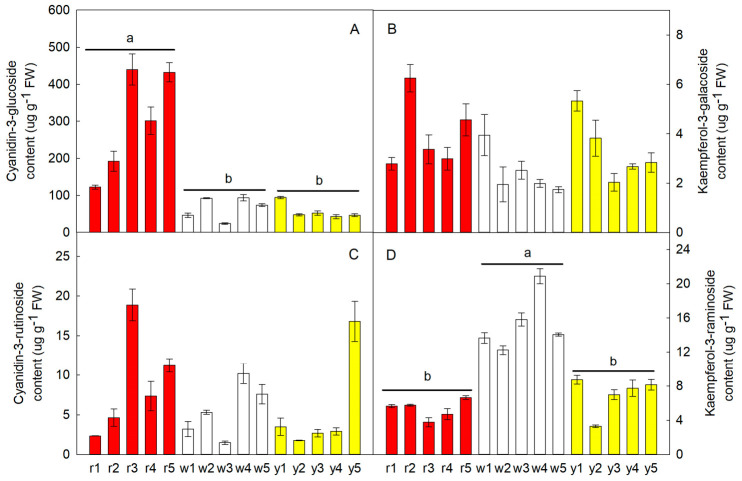
Targeted analysis of anthocyanins and kaempferol glycosides in peach skin from accessions with different flesh colors. (**A**) Cyanidin-3-glucoside content; (**B**) Kaempferol-3-galactoside content; (**C**) Cyanidin-3-rutinoside content; (**D**) Kaempferol-3-rhamnoside content. Different lowercase letters indicate significant differences among color groups (one-way ANOVA followed by Tukey’s HSD test, *p* < 0.05). The horizontal line indicates the mean value of each color group. Sample codes are as defined in Figure 2.

The contents of selected anthocyanins and kaempferol glycosides in the skin of peach fruits with different colors were determined by HPLC (Figure 4). Cyanidin-3-glucoside was the predominant anthocyanin detected in the skin, together with a lower level of cyanidin-3-rutinoside. Cyanidin-3-glucoside content was significantly higher in red-fleshed accessions (r) than in white-fleshed (w) and yellow-fleshed (y) accessions, with r3 exhibiting the highest level (439.89 μg g^−1^ FW), approximately 9.5-fold higher than that of w1 and 4.7-fold higher than that of y1. No significant difference in the average content of this compound was observed between white- and yellow-fleshed accessions (Figure 4A,C). The selected kaempferol glycosides detected or putatively assigned in the skin were kaempferol-3-rhamnoside and kaempferol-3-galactoside. Notably, kaempferol-3-rhamnoside accumulated at significantly higher levels in white-fleshed accessions than in red- and yellow-fleshed accessions, with w4 showing the highest content (20.87 μg g^−1^ FW), approximately 3.7-fold higher than that of r1 and 2.4-fold higher than that of y1. No significant difference in the content of this compound was observed between red- and yellow-fleshed accessions (Figure 4B,D). These results suggest that cyanidin-3-glucoside accumulation is closely associated with red skin pigmentation, whereas kaempferol-3-rhamnoside shows preferential accumulation in white-skinned accessions.

To further verify the consistency between the spectrophotometric determination of total anthocyanins and the HPLC-based quantification of individual anthocyanins, correlation analysis was performed. Total anthocyanin content showed a significant positive correlation with cyanidin-3-glucoside content (r = 0.8048, *p* = 0.0003) and was also positively correlated with cyanidin-3-rutinoside content (r = 0.5700, *p* = 0.0265). These results indicate that the total anthocyanin values were broadly consistent with the HPLC-detected major anthocyanin components, supporting the reliability of the anthocyanin quantification results (Supplementary Figure S3).

### 3.4. Expression Analysis of Key Flavonoid Metabolic Genes in Peach Skin with Different Flesh Colors

**Figure 5:**
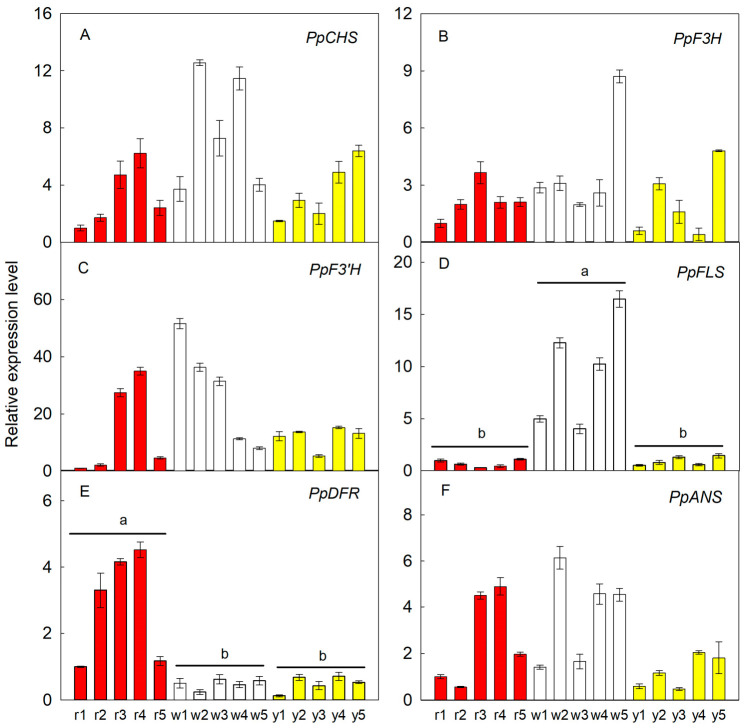
Expression analysis of key genes involved in anthocyanin and kaempferol glycoside metabolism in peach skin. (**A**) *PpCHS*; (**B**) *PpF3H*; (**C**) *PpF3′H*; (**D**) *PpFLS*; (**E**) *PpDFR*; (**F**) *PpANS*. Different lowercase letters indicate significant differences among color groups based on one-way ANOVA followed by Tukey’s HSD test (*p* < 0.05). The horizontal line indicates the mean value of each color group. ns indicates no significant difference. Sample codes are as defined in Figure 2.

To investigate gene expression patterns associated with differential accumulation of anthocyanins and selected kaempferol glycosides, the expression levels of key flavonoid pathway genes (*PpCHS*, *PpF3H*, *PpF3′H*, *PpFLS*, *PpDFR*, and *PpANS*) were analyzed across different color groups (Figure 5). *PpFLS* expression was significantly higher in white-fleshed accessions (w) than in red-fleshed (r) and yellow-fleshed (y) accessions, with w5 exhibiting the highest expression level, approximately 16.5-fold higher than that of r1 (Figure 5D). In contrast, *PpDFR* expression was significantly higher in red-fleshed accessions than in white- and yellow-fleshed accessions, with r4 showing the highest expression level, approximately 18.8-fold higher than that of w2 (Figure 5E). No significant differences in the expression levels of *PpCHS*, *PpF3H*, *PpF3′H*, or *PpANS* were observed among the different color groups based on formal statistical tests. These results indicate that higher *PpDFR* expression in red-fleshed accessions and higher *PpFLS* expression in white-fleshed accessions were associated with preferential accumulation of anthocyanins and kaempferol-3-rhamnoside, respectively.

### 3.5. Functional Analysis of a Key Flavonoid Metabolic Gene from Peach

**Figure 6:**
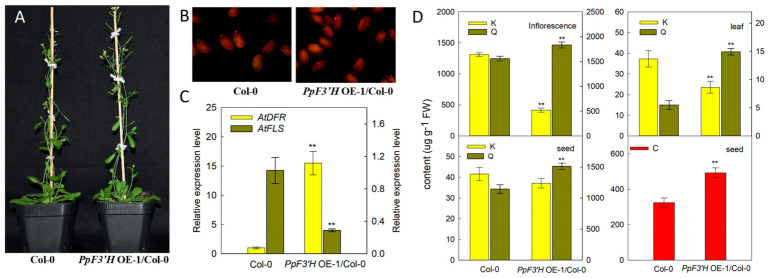
Phenotypic, gene-expression, and flavonoid-content analyses of *Arabidopsis* heterologously overexpressing *PpF3′H*. (**A**) Phenotype of wild-type (Col-0) and *PpF3′H*-overexpressing (OE) *Arabidopsis* plants; (**B**) seed phenotype; (**C**) relative expression of *AtDFR* and *AtFLS*; (**D**) kaempferol-to-quercetin ratio in inflorescences, leaves, and seeds, and total anthocyanin content in seeds. K, kaempferol; Q, quercetin; C, cyanidin. Asterisks indicate significant differences between WT and OE plants (** *p* < 0.01).

To evaluate the potential function of peach *PpF3′H* in flavonoid metabolism, *PpF3′H* was heterologously overexpressed in *Arabidopsis*. As shown in Figure 6A, the overexpression (OE) plants exhibited normal growth, and their seeds displayed a visibly redder phenotype compared with wild-type (Col-0) seeds (Figure 6B). Gene expression analysis showed that *AtDFR* expression was higher in OE plants, while *AtFLS* expression was reduced (Figure 6C). Because *PpF3′H* encodes a catalytic enzyme rather than a transcription factor, these changes in *AtDFR* and *AtFLS* expression should be interpreted as possible indirect consequences of altered flavonoid metabolism or feedback regulation rather than direct transcriptional regulation by *PpF3′H*. Flavonoid content analysis revealed that the kaempferol-to-quercetin ratio was lower in OE plants compared with wild-type plants across inflorescences, leaves, and seeds (Figure 6D). Furthermore, total anthocyanin content in OE seeds was significantly elevated, approximately 1.5-fold higher than that of wild-type seeds (Figure 6D). These results indicate that heterologous expression of *PpF3′H* is associated with altered flavonoid composition and increased anthocyanin accumulation in *Arabidopsis*. However, this experiment should be considered a heterologous functional assay and does not by itself prove that *PpF3′H* is the rate-limiting determinant of peach fruit pigmentation.

### 3.6. Correlation Analysis Between Skin Pigmentation, Target Metabolites, and Gene Expression

**Figure 7:**
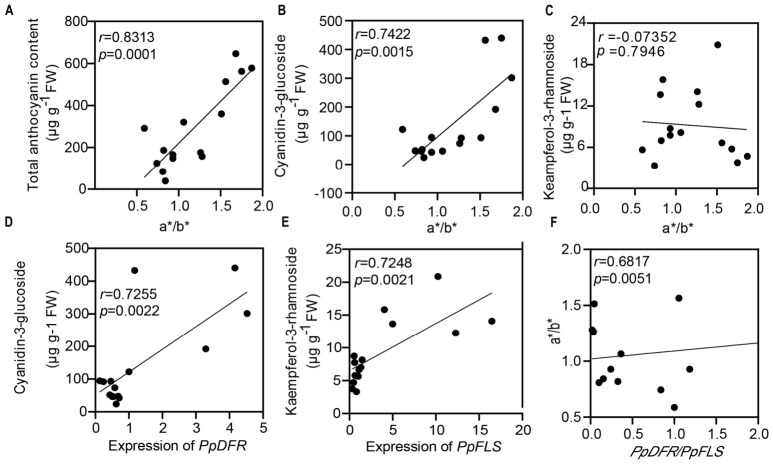
Correlation analysis between peach skin coloration, flavonoid accumulation, and related gene expression. (**A**) Correlation between total anthocyanin content and the a*/b* value. (**B**) Correlation between cyanidin-3-glucoside content and the a*/b* value. (**C**) Correlation between kaempferol-3-rhamnoside content and the a*/b* value. (**D**) Correlation between *PpDFR* expression and cyanidin-3-glucoside content. (**E**) Correlation between *PpFLS* expression and kaempferol-3-rhamnoside content. (**F**) Correlation between the *PpDFR*/*PpFLS* expression ratio and the a*/b* value. Pearson correlation coefficients and *p*-values are shown in each panel. The fitted lines indicate linear regression trends.

To further examine the relationship between peach skin coloration and flavonoid accumulation, correlation analyses were performed among color parameters, anthocyanin/flavonol contents, and the expression levels of related structural genes (Figure 7). Total anthocyanin content was significantly and positively correlated with the a*/b* value (r = 0.8313, *p* = 0.0001) (Figure 7A), and a similar positive correlation was observed between cyanidin-3-glucoside content and the a*/b* value (r = 0.7422, *p* = 0.0015) (Figure 7B), suggesting that anthocyanin accumulation was closely associated with the red coloration of peach skin. In contrast, kaempferol-3-rhamnoside content showed no significant correlation with the a*/b* value (r = −0.07352, *p* = 0.7946) (Figure 7C). Further analysis showed that cyanidin-3-glucoside content was significantly correlated with *PpDFR* expression (r = 0.7255, *p* = 0.0022) (Figure 7D), while kaempferol-3-rhamnoside content was significantly correlated with *PpFLS* expression (r = 0.7248, *p* = 0.0021) (Figure 7E). Moreover, the *PpDFR*/*PpFLS* expression ratio was positively correlated with the a*/b* value (r = 0.6817, *p* = 0.0051) (Figure 7F), indicating that the relative activity of the anthocyanin and flavonol branches may contribute to variation in peach skin coloration.

## 4. Discussion

### 4.1. Targeted Metabolic Traits of Anthocyanins and Kaempferol Glycosides

Cyanidin-3-O-glucoside was confirmed as the major anthocyanin responsible for red skin coloration, consistent with previous reports. Recent multi-omics analyses across multiple blood-fleshed peach varieties further identified cyanidin-3-O-glucoside, together with pelargonidin-3-O-glucoside and total anthocyanin, as the three most critical intrinsic indicators influencing total antioxidant capacity [24]. Red-fleshed accessions had much higher total anthocyanin levels in flesh than white and yellow ones. Trace anthocyanins detected in the white accession ‘Chunmi’ (w5) and yellow accession ‘Xin Zhongnan 11-30’ (y4) suggest leaky expression of the biosynthetic pathway, warranting further study. Similar observations in pear indicate that anthocyanin accumulation is influenced by genotype and environment [25].

Kaempferol-3-rhamnoside preferentially accumulated in white-fleshed accessions, correlating with lower *PpDFR* expression and directing flux toward the FLS-catalyzed flavonol branch. This supports the competitive relationship between anthocyanin and kaempferol glycoside biosynthesis reported in apple [12] and in the alpine flower *Meconopsis integrifolia*, where a high *MiFLS2*/*MiDFR6* expression ratio channels metabolic flux toward flavonol accumulation and confers yellow coloration [26]. Flavonol accumulation in white peach may function in defense, UV protection, and pollen fertility [6,12]. Whether its enrichment relates to breeding history (e.g., emphasis on flavor over color) remains unclear.

### 4.2. Association of PpDFR and PpFLS Expression with Anthocyanin and Kaempferol Glycoside Accumulation

Dihydroflavonols are common precursors for anthocyanins (via DFR) and flavonols (via FLS). Because DFR and FLS use related substrates, their relative activity can influence whether these precursors are associated more strongly with anthocyanin or flavonol accumulation. We found that *PpDFR* was highly expressed in red accessions, while *PpFLS* was highly expressed in white accessions, with no significant differences in *PpCHS*, *PpF3H*, *PpF3′H*, or *PpANS* among groups (Figure 5).

This supports the branch competition hypothesis: flux direction depends on relative DFR/FLS activity, not upstream gene expression. Similar findings exist in blood-fleshed peach [3] and wild *Prunus mira* [27]. Recent evidence from CRISPR/Cas9-mediated DFR knockout in red leaf lettuce demonstrated that redirecting metabolic flux away from anthocyanins leads to altered flavonoid profiles, providing direct genetic evidence for compensatory flux redistribution upon perturbation of one branch [18]. Conversely, in *Meconopsis integrifolia*, *MiFLS2* overexpression increased flavonol accumulation while suppressing anthocyanin biosynthesis, and its expression was negatively correlated with *MiDFR6*, hinting at a possible feedback inhibitory loop between the two branches [26]. These findings collectively suggest that the DFR-FLS competition is embedded in a dynamic regulatory network capable of sensing and adjusting flux distribution in response to genetic perturbations.

The lack of *PpANS* expression differences suggests that variation in anthocyanin accumulation may not be explained by all downstream structural genes equally. One possible explanation is that substrate availability or earlier branch-related steps limit anthocyanin accumulation in white-fleshed accessions, even when *PpANS* transcript levels are not significantly different. In addition, substrate preferences of DFR and FLS may reinforce differences in flavonoid composition [19,28]. Functional characterization of peach DFR and FLS enzyme activity will be needed to test this hypothesis directly.

The correlation analysis further supports this branch-balance model. Skin a*/b* values were positively correlated with total anthocyanin and cyanidin-3-glucoside contents, whereas kaempferol-3-rhamnoside showed no significant association with redness. In addition, cyanidin-3-glucoside and kaempferol-3-rhamnoside were positively correlated with *PpDFR* and *PpFLS* expression, respectively. These results suggest that peach skin redness is mainly associated with anthocyanin accumulation, while kaempferol-3-rhamnoside accumulation is more closely linked to the flavonol branch rather than direct color formation. The positive correlation between the *PpDFR*/*PpFLS* ratio and a*/b* value also indicates that the relative expression balance between these two branch genes may better explain pigmentation variation than either gene alone.

### 4.3. Heterologous PpF3′H Expression Is Associated with Altered Flavonoid Composition

Heterologous overexpression of peach *PpF3′H* in *Arabidopsis* was associated with darker seeds, increased *AtDFR* expression, reduced *AtFLS* expression, a lower kaempferol-to-quercetin ratio, and increased seed anthocyanins (Figure 6). Mechanistically, F3′H catalyzes the conversion of dihydrokaempferol to dihydroquercetin [19,28]. Because DFR generally shows higher catalytic efficiency toward dihydroquercetin than toward dihydrokaempferol, increased dihydroquercetin availability may favor anthocyanin-associated metabolism through DFR [19,28]. Meanwhile, the reduced kaempferol-to-quercetin ratio suggests a shift in flavonol composition toward quercetin-derived compounds. Supporting evidence from other plants has shown coordinated expression of F3′H, DFR, and ANS during red color development in pear [25]. Therefore, *PpF3′H* appears to have catalytic capacity to alter flavonoid composition in a heterologous system. However, because *PpF3′H* transcript abundance did not differ significantly among peach color groups, the differential accumulation of anthocyanins and kaempferol glycosides in peach skin appears more closely associated with *PpDFR* and *PpFLS* expression patterns than with differential *PpF3′H* transcript abundance.

### 4.4. Upstream Regulation of DFR and FLS and the Color Network

Recent studies have identified upstream regulators such as the miR164a-NAC module controlling *PpMYB10.1* expression [29]. These regulators may influence pigmentation through anthocyanin structural genes and broader flavonoid networks. However, because the present study did not directly test upstream regulation of *PpDFR* or *PpFLS*, this possibility is discussed only as a future research direction.

Light signaling also plays an important role. A recent study on the flat peach cultivar ‘Zhongyoupan 9’ revealed that *PpHY5* positively regulates anthocyanin biosynthesis even under artificial darkness, and VIGS-mediated silencing of *PpHY5* resulted in significantly reduced anthocyanin levels, establishing PpHY5 as a key regulator in light-independent anthocyanin accumulation [30]. Environmental factors such as temperature and plant hormones (ABA, ethylene) also influence fruit coloration [20], and post-harvest ethylene management has been shown to improve fruit surface color [31].

### 4.5. Limitations, Conclusions, and Future Perspectives

This study provides targeted metabolite and gene-expression evidence for differential anthocyanin and selected kaempferol glycoside accumulation in peach. Specifically, cyanidin-3-O-glucoside was the major red-associated pigment, kaempferol-3-rhamnoside preferentially accumulated in white-fleshed accessions, and the relative expression patterns of *PpDFR* and *PpFLS* were associated with these contrasting accumulation tendencies. Heterologous expression of *PpF3′H* in *Arabidopsis* further supported its capacity to influence flavonoid composition and anthocyanin accumulation. This experiment is more suitable as preliminary support for the catalytic function of *PpF3′H*, rather than being able to fully exclude insertional site effects. However, this study did not directly measure metabolic flux, enzyme activity, or causal genetic effects in peach; therefore, terms such as pathway redistribution and branch preference should be interpreted as hypotheses supported by metabolite accumulation and expression patterns rather than direct flux evidence. Understanding these associations may help identify candidate markers for breeding peach cultivars with improved pigmentation and flavonoid-related nutritional quality.

Future research should focus on: (1) validating these associations across multiple years, locations, and developmental stages; (2) directly testing *PpDFR*, *PpFLS*, and *PpF3′H* enzyme activities and substrate preferences; (3) applying transient expression, VIGS, or gene-editing approaches in peach to test causal relationships; and (4) integrating upstream regulators, environmental signals, and branch-related enzymes into a more complete model of peach pigmentation. Such studies will be necessary to distinguish correlation from causality and to determine whether manipulation of these genes can predictably improve peach coloration and flavonoid composition.

## Data Availability

The original contributions presented in this study are included in the article/Supplementary Materials. Further inquiries can be directed to the corresponding authors.

## Supplementary Materials

The following supporting information can be downloaded at: https://www.mdpi.com/article/10.3390/foods15122225/s1. Table S1: Fruit quality indices of different peach varieties with contrasting colors. Table S2: Analysis of the color of peaches with white, yellow and red flesh. Table S3: Primers used for real-time quantitative PCR analysis. Figure S1: Representative HPLC chromatograms of peach (accession 6-11-50) peel extracts and corresponding chemical standards. Chromatograms A, C, E, G were acquired at 520 nm for anthocyanin detection; chromatograms B, D, F, H were recorded at 365 nm for kaempferol detection. Figure S2: Comparison and analysis of amino acid sequence similarity of F3′H among peach, tomato and *Arabidopsis thaliana*. Figure S3: Correlation analysis between the content of key anthocyanin components and total anthocyanin content in peach fruit.
