# Supplementary material for: Targeted Metabolite and Gene Expression Analysis of Anthocyanin and Kaempferol Glycoside Accumulation in Peach Accessions with Contrasting Flesh and Skin Pigmentation
Source: Foods. 2026 Jun 20;15(12):2225. doi: 10.3390/foods15122225 (PMC13298405; doi:10.3390/foods15122225)

Supplementary Table S1. Fruit Quality Indexes of Different Peach Varieties with Contrasting Color.

Data include single fruit weight, flesh firmness, fruit dimensions, fruit shape index, soluble solids content (SSC), titratable acidity (TA), and SSC/TA ratio across 15 peach accessions.

Code	Variety	Single Fruit Weight (g)	Flesh Firmness (N/Kg)	Longitudinal Diameter (mm)	Transverse Diameter (mm)	Fruit Shape Index	Soluble Solids Content (%)	Titratable Acidity (%)	Solid-Acid Ratio
r1	7-3-58	146.47±21.86	9.82±1.45	67.59±2.78	65.24±3.24	1.04±0.02	7.9±0.53	0.27±0.01	29.28±2.08
r2	7-3-64	125.52±17.87	5.7±0.19	60.53±1.23	64.15±4.57	0.95±0.07	8.03±0.23	0.21±0.01	38.34±2.86
r3	6-11-50	126.61±24.87	4.85±0.67	62.78±5.72	62.94±6.84	1.0±0.13	9.9±0.87	0.15±0.01	66.43±10.09
r4	Bei 22-31 Dong	92.45±13.76	4.02±0.46	56.94±2.02	56.46±4.07	1.01±0.05	12.4±1.78	0.23±0.01	54.14±9.37
r5	Bei 23-20 Xi	113.69±20.95	2.41±0.17	58.17±3.13	59.39±4.28	0.98±0.02	7.57±1.11	0.38±0.01	19.95±3.2
w1	08 Bei-8-4	122.55±10.28	8.87±0.54	64.98±2.67	60.47±2.5	1.07±0.05	9.03±0.72	0.27±0.01	33.49±3.09
w2	Zhongyou Mini No.1	104.27±8.43	7.15±1.2	56.65±3.39	58.29±0.65	0.97±0.06	16.37±2.2	0.52±0.01	31.51±4.59
w3	Zhongyou 12	125.78±11.46	5.14±0.84	56.56±1.5	60.76±4.71	0.93±0.07	10.83±1.16	0.26±0.01	41.59±2.9
w4	Xin Zhongnan 40-35	235.75±42.69	7.5±0.34	71.27±4.64	79.07±7.8	0.9±0.05	8.57±0.85	0.26±0.01	32.9±2.01
w5	Chunmi	183.79±29.87	4.49±0.7	71.47±4.22	69.08±2.82	1.03±0.06	7.63±0.51	0.26±0.01	29.42±2.92
y1	Xin Xibei 57-61	130.17±19.55	6.66±0.19	57.29±4.31	60.49±1.1	0.95±0.06	7.27±0.75	0.3±0.01	24.27±3.02
y2	Zhongyou 19	205.56±12.08	9.79±1.19	66.97±1.82	76.63±5.25	0.87±0.04	14.57±2.22	0.33±0.01	44.21±7.3
y3	Xin Xinan 34-8	160.62±47.56	10.02±0.58	63.71±5.94	67.34±4.81	0.94±0.03	8.03±0.74	0.33±0.01	24.4±2.92
y4	Xin Zhongnan 11-30	114.0±22.08	6.9±1.11	55.48±0.94	58.22±3.67	0.96±0.06	9.6±0.61	0.27±0.01	35.64±3.37
y5	Huangjinmi No.7	95.67±21.34	7.02±0.64	57.68±3.68	57.24±7.33	1.01±0.07	10.03±1.07	0.25±0.01	40.28±5.67

Supplementary Table S2. Analysis of Color of Peaches with White, Yellow and Red Flesh

Code	Variety	Replicate 1	Replicate 2	Replicate 3
r1	7-3-58	L=48.83;a=17.01;b=22.73	L=66.68;a=14.17;b=25.92	L=70.13;a=11.83;b=24.58
r2	7-3-64	L=47.29;a=37.43;b=21.13	L=48.75;a=32.47;b=21.69	L=46.99;a=34.40;b=19.40
r3	6-11-50	L=41.46;a=29.57;b=15.33	L=42.68;a=31.75;b=17.23	L=48.53;a=29.23;b=19.16
r4	Bei 22-31 Dong	L=30.60;a=26.95;b=10.16	L=44.34;a=35.45;b=22.92	L=37.57;a=35.24;b=19.02
r5	Bei 23-20 Xi	L=46.56;a=23.47;b=17.32	L=42.69;a=22.83;b=15.25	L=42.37;a=30.83;b=16.71
w1	08 Bei-8-4	L=75.03;a=11.80;b=21.62	L=64.79;a=23.44;b=21.90	L=71.89;a=17.22;b=21.42
w2	Zhongyou Mini No.1	L=46.33;a=35.28;b=23.37	L=61.10;a=26.62;b=24.26	L=54.61;a=35.04;b=28.05
w3	Zhongyou 12	L=50.27;a=31.28;b=25.45	L=50.98;a=26.20;b=27.56	L=59.47;a=12.50;b=30.09
w4	Xin Zhongnan 40-35	L=45.69;a=29.99;b=19.58	L=43.60;a=28.55;b=18.89	L=42.11;a=23.96;b=16.01
w5	Chunmi	L=51.60;a=33.37;b=26.71	L=59.07;a=29.18;b=26.34	L=46.61;a=32.74;b=22.32
y1	Xin Xibei 57-61	L=48.36;a=28.32;b=25.92	L=59.21;a=21.09;b=40.97	L=37.97;a=24.76;b=13.11
y2	Zhongyou 19	L=45.01;a=38.44;b=29.62	L=72.00;a=16.37;b=55.77	L=52.20;a=38.50;b=40.09
y3	Xin Xinan 34-8	L=52.61;a=24.71;b=33.20	L=58.02;a=18.74;b=35.45	L=45.20;a=32.39;b=24.02
y4	Xin Zhongnan 11-30	L=54.60;a=28.90;b=34.51	L=46.52;a=34.05;b=36.01	L=49.17;a=31.11;b=30.88
y5	Huangjinmi No.7	L=39.65;a=33.68;b=20.74	L=60.30;a=27.94;b=43.64	L=44.23;a=33.75;b=25.16

Supplementary Table S3. Primers used for real-time quantitative PCR analysis.

Gene name	GenBank	Forward sequence (5'-3')	Reverse sequence (5'-3')	Amplicon size	Efficiency
PpCHS	KX823936.1	CAACTGAAGGCACACCCACT	TAGGTCTCCCCCAGTTCTTG	111 bp	94.3%
PpF3H	HM543570.1	TGACCCAGGCACAATTACCC	AGCCTGCTGCTGTTTGAGTT	204 bp	101.2%
PpF3'H	JQ697494.1	TTGAGTTCCGACCCGAAAGG	GGTTAGCCCATCAGCCAAGG	197 bp	98.7%
PpFLS	KP050782.1	CTTGCTCTTGGTGTGGTTGC	CATTGACGAGCTTGGGGGTGA	292 bp	100.6%
PpDFR	HM543571.1	AAACCGACTGGAGCGATGTT	GGCAGAGGTCGTCCAAGTGA	276 bp	97.8%
PpANS	KJ484547.1	CTTGCACTTGGTGTCGAAGC	TGGCCCACTTGCCTTCATAG	103 bp	99.1%
PpActin	XM_007211382.2	TGCGAGGTTGGCTTGGTTAT	AGAAGGAAACACAGCCCTGG	172 bp	100.5%
AtDFR	NM_123645.4	ACGTGGTTACTTTGTTCGTGCCA	CCGTTTATGGCATCATCGTAGC	150 bp	96.3%
AtFLS	NM_001203337.1	TCACAACATTCCGAGGTCCAA	CTTCGTCGGGATCGCTTAGA	63 bp	103.3%
AtActin2	NM_112764.4	CACGAGGGAAAAGGCTGTCT	CCGGAGATTCAAAACGGCTG	210 bp	102.7%

Figure S1 HPLC Representative HPLC chromatograms of peach (accession 6-11-50) peel extracts and corresponding chemical standards. Chromatograms A, C, D, G were acquired at 520 nm for anthocyanin detection; chromatograms B, E, F, H were recorded at 365 nm for kaempferol detection.

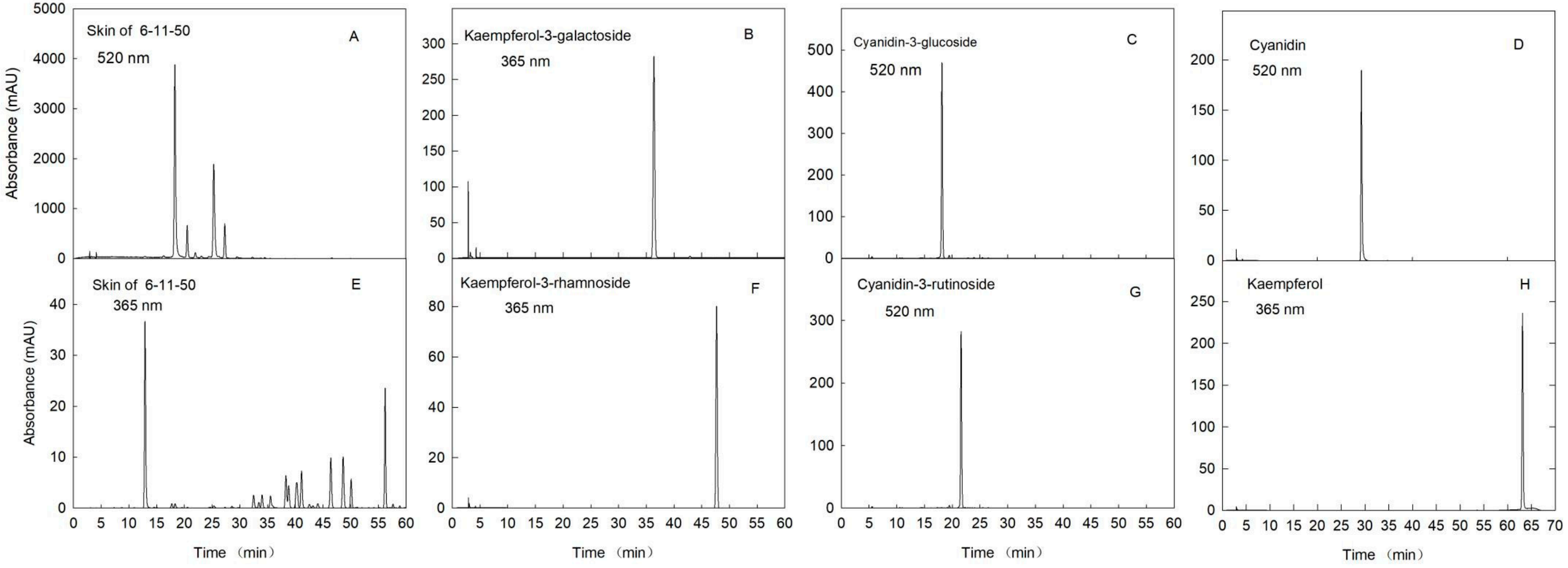

Supplementary Figure S2 Comparison and Analysis of Amino Acid Sequence Similarity of F3'H among Peach, Tomato and *Arabidopsis thaliana.*

```
SlF3'H    MAIFFLILYTIISSIILHFSLSLFFRKRYPVPLPPGPKPWPIIGNIIQLGPKPHQSTASMARTYGPLMHLRMGFVDVVVA   80
AtF3'H    MATLFLTILLATVLFLILRIFSHRRNRSHNNRLPPGPNPWPIIGNLPHMGTKPHRTLSAMVTTYGPILHLRLGFVDVVVA   80
PpF3'H    ..MFILIFITVVFAALLYRLLFS..GNRHSLPLPPGPKPWPIVGNLPHLGPVPHHSLAALARQYGPLMHLRLGFVDVVVA   76
Consensus    l lppgp pwpi gn g ph ygp hlr gfvdvvva

SlF3'H    ASASVAAQFLKNHDANFSSRPPNSGAKHMAYNYHDLVFAPYGPRWRMLRKICSVHLFSTKALDDFRHVRQEEVRTLTRAL   160
AtF3'H    ASKSVAEQFLKIHDANFASRPPNSGAKHMAYNYQDLVFAPYGHRWRLLRKISSVHLFSAKALEDFKHVRQEEVGTLTREL   160
PpF3'H    ASASVASQFLKTHDTNFSSRPPNSGAKHLAYNYHDLVFAPYGPRWRMLRKISSVHLFSGKALDDLRHVRQEEVAVLAHGL   156
Consensusas sva qflk hd nf srppnsgakh ayny dlvfapyg rwr lrki svhlfs kal d hvrqeev l l

SlF3'H    ANAGQNPIKLGQLLNVCTTNALARVMLGKRVFADGTNGMDPQAEEFKLMVVEMMVLAGVFNIGDFIPALDWMDIQGVAGK   240
AtF3'H    VRVGTKPVNLGQLVNMCVVNALGREMIGRRLFG...ADADHKADEFRSMVTEMMALAGVFNIGDFVPSLDWLDLQGVAGK   237
PpF3'H    AGAGSKPVNLAQLLNVCTVNALGRVMVGKRLFGDGSGSGDEKADEFKEMVVEMMVLAGVFNIGDFIPALEWLDLQGVAAK   236
Consensus   g p l ql n c nal r m g r f d a ef mv emm lagvfnigdf p l w d qgva k

SlF3'H    MKKLHARFDAFLTTILEEHKEKRVGESKEQGDLLNTLISLKNEEDDN.GGKLTDTEIKALLLNLFLAGTDTSSSTVEWAI   319
AtF3'H    MKRLHKRFDAFLSSILKEHEMN..GQDQKHTDMLSTLISLKGTDLDGDGGSLTDTEIKALLLNMFTAGTDTSASTVDWAI   315
PpF3'H    MKKLHKRFDAFLTAIVEEHKKS...SGGKHGDMLTTLLSLK.EDADGEGAKLTDTEIKALLLNMFTAGTDTSSSTVEWAI   312
Consensusmk lh rfdafl i eh d l tl slk d g ltdteikallln f agtdts stv wai

SlF3'H    AELIRNPKILAQAQQEIDKVVGKNRLVMESDLAQLTYLEAIVKETFRLHPSTPLSLPRIASESCEINGYFIPKGSTLLVN   399
AtF3'H    AELIRHPDIMVKAQEELDIVVGRDRPVNESDIAQLPYLQAVIKENFRLHPPTPLSLPHIASESCEINGYHIPKGSTLLTN   395
PpF3'H    AELIRHPKILAQVQQELDQVVGRDQLVTELDLPNLTYLQAVIKETFRLHPSTPLSLPRMASESCEINSFHIPKGATLLVN   392
Consensusael r p i q e d vvg v e d l yl a ke frlhp tplslp asescein ipkg tll n

SlF3'H    VWAIARDPNQWADPIEFRPERFLPGGEKPKVDVKGNDFEVIPFGAGRRICPGMSLGIRMVQLMTATLIHSFNWALPTGQL   479
AtF3'H    IWAIARDPDQWSDPLAFKPERFLPGGEKSGVDVKGSDFELIPFGAGRRICAGLSLGLRTIQFLTATLVQGFDWELAGGVT   475
PpF3'H    VWAISRDPEQWKEPLEFRPERFLPGGEKPHVDVRGNDFEVIPFGAGRRICAGMSLGIRMVHLMAATLVHAFDWTLADGLT   472
Consensus wai rdp qw p f perflpggek vdv g dfe ipfgagrric g slg r atl f w l g

SlF3'H    PDKLNMEEAFGLTLQRADPLVVHPIPRLEAQVYGG..   514
AtF3'H    PEKLNMEESYGLTLQRAVPLVVHPKPRLAPNVYGLGS   512
PpF3'H    PEKLNMDEAYGLTLQRAAPLMVHPRTRLAPHAYEASS   509
Consensusp klnm e gltlqra pl vhp rl y
```

Supplementary Figure S3 Correlation analysis between the content of key anthocyanin components and total anthocyanin content in peach fruit.
(A) Positive correlation between cyanidin-3-glucoside content and total anthocyanin content (*r*=0.8046, *p*=0.003). (B) Positive correlation between cyanidin-3-rutinoside content and total anthocyanin content (*r*=0.5700, *p*=0.0265). FW, fresh weight.

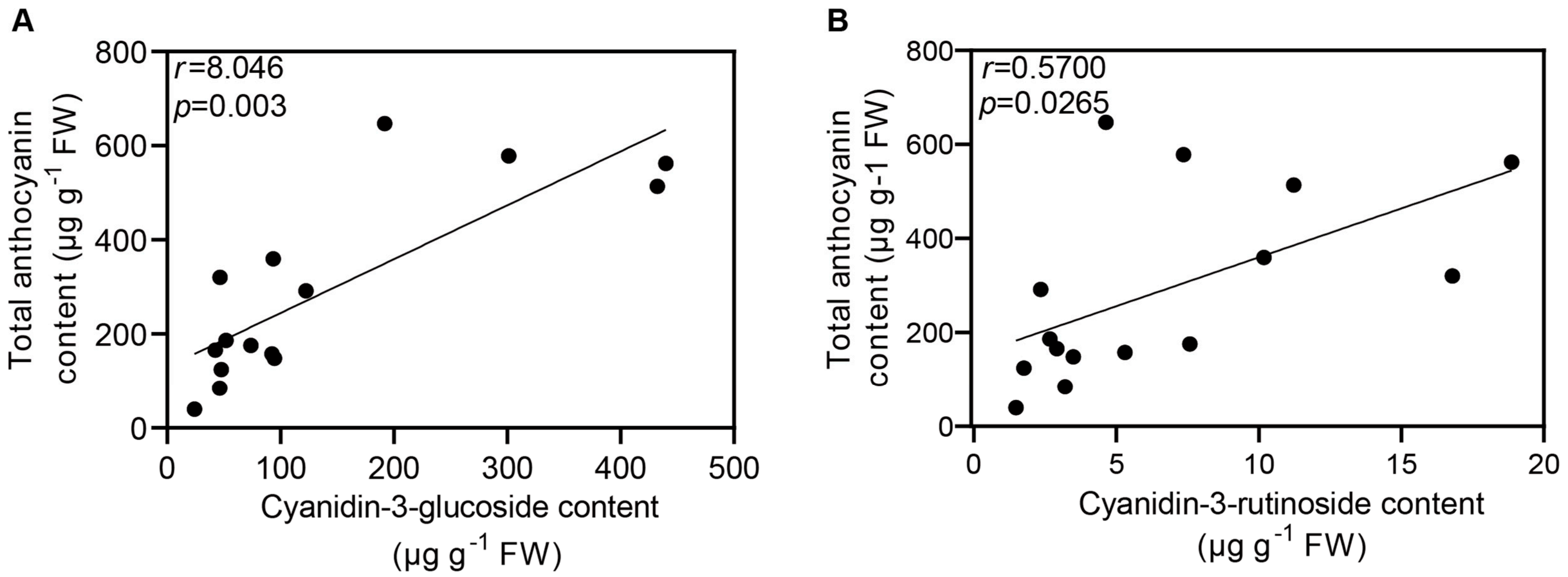

Supplement: Supplementary file 1 [file foods-15-02225-s001.zip › foods-4354505-supplementary.pdf]
